# A Curious Case of a Child With Recurrent Twisting Movements of Limbs

**DOI:** 10.7759/cureus.42037

**Published:** 2023-07-17

**Authors:** Mukund Agrawal, Rameshwar N Chaurasia, Anand Kumar, Abhishek Pathak, Varun K Singh

**Affiliations:** 1 Department of Neurology, Institute of Medical Sciences, Banaras Hindu University, Varanasi, IND

**Keywords:** pediatric movement disorder, epilepsy, psychogenic non-epileptic seizure (pnes), choreiform movement, paroxysmal kinesigenic dyskinesia

## Abstract

Paroxysmal kinesigenic dyskinesia (PKD) is characterized by recurrent attacks of abnormal involuntary movements that are triggered by sudden movement, intention to move, or acceleration. A 10-year-old boy presented with paroxysmal, involuntary twisting movements of the left upper and lower limbs, precipitated by sudden body movements, lasting for 10-15 seconds and subsiding spontaneously. On examination, choreiform movements were observed, which were precipitated by sudden movements during some activities. The patient responded to carbamazepine with complete subsidence of the movements. The diagnosis of PKD was further confirmed by genetic testing. A high suspicion index helps in the prompt and early diagnosis of this rare entity.

## Introduction

Paroxysmal kinesigenic dyskinesia (PKD) is a rare neurologic disorder typified by recurrent attacks of abnormal involuntary movements that are triggered by sudden movement, intention to move, or acceleration [1]. Manifestations can be dystonia (most common), chorea, ballism, athetosis, or a combination of these. Usually, multiple attacks occur every day, ranging from seconds to a minute, and can be unilateral or bilateral [2]. Many patients experience an abnormal sensation such as numbness or ‘‘pins and needles’’ in the affected limb or epigastrium before the attacks [2,3]. An inadequate clinical evaluation may lead to a misdiagnosis of epilepsy or functional illness in this group of disorders [4]. We are reporting an interesting case of a boy with paroxysmal involuntary movements who had a history of treatment as a case of epilepsy/psychogenic non-epileptic seizure (PNES) by different primary care physicians and was later diagnosed as a case of PKD on genetic evaluation.

## Case presentation

A 10-year-old boy born out of non-consanguineous marriage with normal birth and developmental history presented to us with complaints of recurrent, paroxysmal episodes of involuntary twisting movements of the left upper and lower limbs for five months. Initially, these movements occurred only occasionally while running, and the frequency was once every four to five days. After around two months, the frequency increased to 15-20 episodes per day. In the beginning, the movement was restricted to the left upper limb, but later on, the left lower limb also got involved. Initially, these movements appeared only while doing vigorous activities like running and playing, lasted for 10-15 seconds, and subsided spontaneously. Later on, they appeared even while getting up from a sitting position. The movements occurred at a similar frequency irrespective of the state of the patient, whether relaxed and alone, being observed by others, or involved in activities requiring concentration. These events never occurred at rest or sleep; they were not preceded by premonitory sensation; and there was no diurnal variation. The patient also did not have voluntary control over the movements. The child was normal in between these episodes. There was no history of any preceding fever, rashes, breathlessness, or joint pain. There was no associated pain, tonic-clonic movements of limbs, frothing from the mouth, facial twitching, emotional lability, deterioration in scholastic performance, weakness of limbs, slowness in activities, or altered behavior. Sometimes even parents thought that the child was doing these movements voluntarily. He also received antiepileptics (valproate 600 mg/day and clobazam 10 mg/day) and antipsychotic (escitalopram 5 mg/day) medications from different primary care physicians considering the differential diagnosis of epilepsy and PNES, respectively, but there was no clinical response. Family history was negative for similar illnesses in other family members.

On general examination, vitals were stable. His weight was 28 kg. Slit lamp examination was negative for the Kayser-Fleischer ring. Cardiovascular, respiratory, and abdominal examinations were unremarkable. On neurological examination, the patient had precipitation of choreiform movements in the left upper and lower limbs when he stood up from the chair to walk after sitting quietly for 20 minutes (Video 1). The rest of the neurological examination was within normal limits. Based on the history and clinical examination, a provisional diagnosis of PKD was made.

**Video 1:** Video showing the choreiform movements of the left upper and lower limbs that got precipitated on getting up from the chair for walking after sitting quietly for 20 minutes.

­His laboratory investigations such as complete blood count, liver and renal function tests, thyroid function tests, serum calcium, erythrocyte sedimentation rate (ESR), C-reactive protein (CRP), and antistreptolysin O (ASO) titer were normal. Serum ceruloplasmin (29 mg/dl) and 24-hour urinary copper levels (16 µg/24 hours) were within normal limits. Electroencephalography (EEG) and magnetic resonance imaging (MRI) brain were also unremarkable.

His clinical exome sequencing was sent. He was started on a tablet of carbamazepine 100 mg twice daily (7 mg/kg/day) considering the provisional diagnosis of PKD. After 24 hours, he reported no abnormal movements despite following the normal daily routine and physical activities. Clinical exome sequencing revealed a heterozygous mutation in the PRRT 2 gene-c.649dup p.(Arg217ProfsTer8), confirming the diagnosis of PKD. The prompt response to carbamazepine persisted at the 45-day follow-up visit, and the patient also resumed his extracurricular activities in a joyful mood.

## Discussion

The present case was a boy with episodic, paroxysmal choreiform movements of the left upper and lower limbs for five months. The examination was unremarkable except for the abnormal involuntary movements. MRI brain scan was also normal. The genetic evaluation revealed a heterozygous mutation in the PRRT2 gene-c.649dup p.(Arg217ProfsTer8). The PRRT2 gene mutation is pathogenic and the most common mutation causing PKD.

The age of onset in PKD is generally between seven and 15 years, but it has been reported up to 40 years. In the sporadic form, there is a higher male prevalence (4:1) [5]. The majority of cases are idiopathic or familial, while secondary PKD accounts for only a minority of cases. Our patient was a 10-year-old boy with a negative family history.

Recently, Erro et al. [6] proposed a new classification scheme for primary paroxysmal dyskinesias. It consists of two axes: axis I (clinical features) and axis II (genetic determinants) (Table 1).

**Table 1:** Classification of primary paroxysmal dyskinesias. PKD: paroxysmal kinesigenic dyskinesia; PNKD: paroxysmal non-kinesigenic dyskinesia; PED: paroxysmal exercise-induced dyskinesia. Source: Erro et al. [6].

Axis I: Clinical characteristics
A) Inclusion criteria (1 plus one of 2a, b, or c) 1. Paroxysmal attacks of dystonia, chorea, ballism (or a mixture of those) with sudden onset and variable duration (seconds to hours). 2. Paroxysmal dyskinesia is categorized according to the “trigger factor” into one of the following: a. PKD: attacks are triggered by sudden movements, acceleration, or intention to move. b. PNKD: attacks are triggered by coffee, alcohol, and other non-kinesigenic precipitants. c. PED: attacks are triggered by prolonged exercise. B) Exclusion criteria (both 1 and 2) 1. Symptoms are due to another neurological condition. 2. Symptoms are psychogenic.
Axis II: Genetic characteristics
1. Mutations confirmed in one of the known genes (i.e., *PRRT2*, *MR-1*, *KCNMA1*, *SLC2A1*). 2. No mutations in one of the known genes or genetic testing has not been performed (undetermined forms).

The investigations in the present case were unremarkable except for the heterozygous mutation in PRRT2, which is associated with PKD [7]. PRRT2 is inherited in an autosomal dominant fashion with variable penetrance. Mutations in it also cause infantile convulsions and choreoathetosis and benign familial infantile epilepsy [8].

These movement disorders are a common cause of misdiagnosis among clinicians. Sutar et al. also reported a young man with PKD who had received the diagnosis of dissociative disorder, myoclonic epilepsy, panic disorder, and complex partial epilepsy [4].

PKD usually responds well to antiepileptic drugs (AEDs) [1]. The first line of treatment is carbamazepine. The other options are oxcarbazepine, phenytoin, levetiracetam, sodium valproate, topiramate, and lamotrigine [9,10]. The present case responded to carbamazepine but not to valproate with complete subsidence of movements after 24 hours.

## Conclusions

PKD may lead to diagnostic dilemmas and misdiagnosis because of its rarity as well as the lack of awareness among clinicians. Paroxysmal abnormal movements, short duration, precipitating factors like sudden movement, intention to move or acceleration, young age, and positive family history are a few pointers that help in diagnosing this rare entity.
